# Protein Expression of TLR2, TLR4, and TLR9 on Monocytes in TB, HIV, and TB/HIV

**DOI:** 10.1155/2024/9399524

**Published:** 2024-04-17

**Authors:** Wegene Tamene, Liya Wassie, Vincent C. Marconi, Meseret Abebe, Amha Kebede, Ulrich Sack, Rawleigh Howe

**Affiliations:** ^1^HIV and TB Research Directorate, Ethiopian Public Health Institute (EPHI), Addis Ababa, Ethiopia; ^2^Mycobacterial Disease Research Directorate, Armauer Hansen Research Institute (AHRI), Addis Ababa, Ethiopia; ^3^Institute of Clinical Immunology, Medical Faculty, University of Leipzig, Leipzig, Germany; ^4^School of Medicine, Rollins School of Public Health and the Emory Vaccine Center, Emory University, Atlanta, GA, USA

## Abstract

Toll-like receptors (TLRs) have a critical role in recognizing pathogenic patterns and initiating immune responses against TB and HIV. Previously, studies described the gene expression of TLRs in patients with TB and HIV. Here, we demonstrated TLRs protein expressions and their association with clinical status and plasma markers in TB, HIV, and TB/HIV coinfection. The phenotyping of TLR2, TLR4, and TLR9 on CD14+ monocytes and their subsets were determined by multicolor flow cytometry. Host plasma biomarkers and microbial indices were measured using Luminex Multiplex assay and standard of care tools, respectively. TLR2 expression significantly enhanced in TB, slightly increased in HIV but slightly reduced in TB/HIV coinfection compared to apparently health controls (HC). On the other hand, TLR4 expression was significantly increased in TB, HIV, and TB/HIV compared to HC. Expression of TLR4 was equally enhanced on classical and intermediate monocytes while higher TLR2 expression on intermediate than classical monocytes. TLR4 had a positive correlation pattern with plasma biomarkers while TLR2 had an inverse correlation pattern. TLR4 is associated with disease severity while TLR2 is with the immune-competent status of patients. Our findings demonstrated that the pattern of TLR expression is disease as well as monocyte subset specific and distinct factors drive these differences.

## 1. Introduction

Recognition of pathogen-associated molecular patterns (PAMPs) by pattern recognition receptors (PRRs) such as toll-like receptors (TLRs) is the initial stage of a complex immune response. TLR's activation initiates cascade of reactions and results in the expression of inflammatory cytokines, chemokines, and costimulatory receptors [1–3].

To date, 10 human TLRs (TLR1-10) are recognized and they are classified into two subfamilies based on their localization in the cells [4]. TLR1, 2, 4, 5, 6, and 10 are cell surface TLRs which recognize pathogenic proteins, lipids, and lipoproteins while TLR3, 7, 8, and 9 are intracellular TLRs that recognize foreign or damaged nucleic acids [1]. Lipoarabinomannan (LAM), Lipomannen, 19 kDa Lipoprotein, early secreted antigen target 6 (ESAT-6), 38-kDa glycolipid, heat shock protein 65, 50s ribosomal protein and small oligonucleotides are some of *Mycobacterium tuberculosis* (*M.tb*) PAMPs [5, 6]. HIV envelop glycoproteins (gp41 and gp120), core proteins (p17 and p24), and regulatory proteins are some of the HIV PAMPs recognized by TLRs [7].

TLR-mediated *M.tb* elimination involves several processes such as macrophage activation, cytokine production, and immune cell maturation [7, 8]. Previous studies reported alteration of TLR2, TLR3, TLR4, TLR7, TLR8, and TLR9 levels in TB and HIV diseases [7, 9]. TLR2 knockout mice failed to form granuloma and control *M.tb* infection compared to wild-type mice [10, 11]. Similarly, TLR2 and TLR9 knockout mice succumb to *M.tb* infection early compared to wild-type mice [12]. TLR2 and TLR4 synergize to mediate the apoptosis of *M.tb* infected macrophages. TLR4-dependent signaling mediates the balance between apoptotic versus necrotic death of *M.tb* infected cells [13]. TLR7 and TLR9 upregulate the expression of MHC II that activates *M.tb* specific CD4+ T-cells [14]. TLR9 induced type I interferons (IFNs) that facilitate the maturation of anti-*M.tb* CD8+ T cells [15] and immunity against HIV [16]. Similarly, TLR2 gene is also associated with elevated risk of HIV infection and progression [17, 18].

Conversely, TLR2 expressing cells showed considerably high levels of HIV proviral DNA compared to cells lacking the receptor partly due to TLR2 induced CCR5, a coreceptor of HIV, that facilitates infectivity [19]. HIV hinders the production of type I IFN, a potent antiviral, by impairing dendritic cells while increasing IL10, anti-inflammatory cytokine, which dampens protection [16, 20, 21]. Likewise, TLR9-dependent increase in production of IL10 by regulatory T cell hindered immunity against *M.tb* [22]. Blocking TLR9 averted IL10 production and resulted a surge in type I IFN production. Alternately, *M.tb* antigen such as ESAT-6 can directly bind to TLR2 on macrophages and suppress TLR activation [23].

In general, the role and expression of TLRs in TB and HIV are controversial, which is further complicated by the presence of dual infection. Most of the previous studies on TLRs in TB and HIV were based either on animal studies, *in vitro* inductions, or transcriptional profiling. None of the previous studies described TLR expression on monocytes in *ex vivo* settings in patients with HIV or TB/HIV coinfection and there was only one study in patients with TB [24]. In addition, none of the previous studies examined the expression difference of TLRs on monocyte subsets in our patient cohorts. Thus, we described the protein expression of TLRs on monocytes and their subsets in patients with HIV, TB, and TB/HIV coinfection. Furthermore, we assessed the association of microbial indices and host plasma biomarkers with TLRs expression.

## 2. Methods

### 2.1. Study Site and Patient Population

A total of 120 study participants were recruited from health facilities in Addis Ababa, Ethiopia. The study cohorts were as follows: 34 participants with TB but without HIV infection (TB), 35 participants with HIV but without TB infection (HIV), 12 patients with both TB and HIV infections (TB/HIV), and 39 healthy controls (HC) with neither TB nor HIV. Individuals with a previous history of TB disease, autoimmune disorders, chronic diseases (diabetes mellitus, hypertension, cancer, and heart diseases), pregnant women, and those on immunosuppressive medications were excluded. TB diagnosis was made either with bacteriological and/or combined clinical and radiological approaches. HIV was diagnosed using rapid HIV tests based on the Ethiopian national HIV testing algorithm. Neither people in TB nor in HIV groups were on respective treatments while some of the people with TB and HIV coinfection were on antiretroviral therapy (ART). HC was defined as having HIV-negative serostatus and with no clinical sign of active TB disease.

### 2.2. Peripheral Blood Mononuclear Cells (PBMCs) Preparation

Twenty milliliters (ml) of heparinized venous blood were collected, briefly centrifuged, plasma separated then packed cells processed for PBMCs isolation. Each aliquot of packed cells was diluted with equal volume of Roswell Park Memorial Institute (RPMI) media (Sigma), layered over Ficoll-Paque plus on Leucosep tubes (Greiner), and PBMCs separated by density gradient centrifugation. Cells in the supernatant were harvested and washed three times with phosphate-buffered saline. Finally, cells were resuspended in 1 ml RPMI and manually counted on a hemocytometer after staining with trypan blue to check viability of the cells. Dead cells appeared blue as the trypan blue passed through the damaged cell membrane while viable cells were colorless. The average viability of cells was 98%.

### 2.3. Immunophenotyping Using Flow Cytometry

One million freshly isolated PBMCs each was dispensed to the experiment and isotype control tubes. Anti-CD14 PE and CD16 APC-H7 (BD bioscience), TLR2 Alexa Flour 647 and TLR4 Brilliant violet 421 (Biolegend) were added to the experimental tube while anti-CD14 PE, CD16 APC-H7, IgG2a Alexa Flour 647, and IgG2a Brilliant violet 421 added to the isotype control tubes then cells were incubated for 20 min in dark place to stain the surface markers. At the end of incubation, cells were washed once with FACS buffer (PBS containing 1 mM EDTA and 0.1% bovine serum albumin). Surface stained cells were then fixed with 0.5 ml of 1% paraformaldehyde for 20 min at 4°C. After washing the cells once with FACS buffer, 1 ml of cold 1x Perm-2 (BD, USA) was added to permeabilized cells to proceed to intracellular staining. Permeabilized cells washed once with cold FACS buffer, anti-TLR9 Alexa flour 488 (R&D systems), and sheep IgG Alexa flour 488 were added to the experimental and isotype control tubes, respectively. Cells were incubated for 20 min at 4°C and washed once with FACS buffer at the end of incubation. For final fixation step, 0.5 ml of 4% paraformaldehyde was added to the cells, incubated for 20 min at 4°C. Finally, stained cells were washed and made ready for acquisition.

FACSCanto II (BD bioscience) cytometer was used to acquire data using FACSDiva software (BD bioscience). Raw data collected on FACSDiva software analyzed using Flowjo 9.4.6 Software (FlowJo, USA). FL8 channel was used as a damp channel to detect and eliminate nonspecific fluorescing cells. Cells positive for FL8 were gated out as no flourochrome staining was included to be read in this channel. Doublets were gated out by forward light scatter (FSC)-height and FSC-area plot. CD14+ monocyte-enriched populations were gated based on CD14 positivity. Total CD14+ monocytes were further subclassified in to classical (CM) and intermediate (IM) monocytes subsets on the basis of CD16 positivity. Then, the median fluorescence intensity (MFI) of TLR2, TLR4, and TLR9 measured on total CD14+ monocytes as well as CM and IM subsets. Finally, net MFI (nMFI) of TLRs was calculated by subtracting the MFI of appropriate isotype control from MFI of corresponding TLR antibody (*Supplementary 1*).

### 2.4. Plasma Cytokine Analysis

Production of inflammatory cytokines and chemokines are some of the downstream outputs of TLR signaling pathways. In chronic inflammatory diseases such as TB or HIV, TLRs are continuously activated. Thus, we measured plasma biomarkers using human premixed, multianalyte kit (R&D, Germany) according to the manufacturer's instructions (R&D systems). Thawed plasma specimens were briefly centrifuged, and the supernatant was transferred to new 1.5 ml Eppendorf tubes. Specimens were then diluted twofold with buffer and dispensed to the reaction plates. Specimens and standards were tested in duplicate. The mean coefficient of variation (CV) between duplicates was 8% (ranging 4%–10%). Internal luminex assay controls (low and high) were used as an internal quality control check. All samples were tested using same lot numbered reagents. Standards and samples were acquired on MAGPIX Luminex machine (xMAP Tech). Machine red mean fluorescence intensity (MFI) of biomarkers was converted to concentration in reference to the standard curve generated using xPONANT v4.2 software. A panel of fourteen inflammatory chemokines and cytokines: CCL2, CCL3, CCL4, CCL7, CXCL10 (IP10), IFN*γ*, IFN*α*, IL1*α*, IL1*β*, TNF*α*, IL6, IL12p70, IL17, and IL10 were included in the plasma panel. The cytokines included in this study were selected based on their relevance to TB and HIV infections [25].

### 2.5. TB Diagnosis

At the TB clinic, patients suspected of having TB initially screened by clinical signs and symptoms. Then, sputum samples collected from these patients were assessed for *M.tb* bacteriologically, either by acid-fast bacilli (AFB) smear microscopy and/or GeneXpert MTB/RIF molecular assay (Cepheid). Bacteriologically negative TB cases were diagnosed by clinical sign and chest X-ray. Before start of anti-TB treatment, each participant gave sputum sample for further characterization at the National TB reference laboratory. At the National TB reference laboratory, sputum samples were cultured on Lowenstein Jensen (LJ) solid and mycobacteria growth indicator tube (MGIT) 9200 liquid culture media. *M.tb* species identification was made using a rapid test kit. Urinary LAM was assessed using determine LAM Ag lateral flow assay (Alere). Semiquantitative visual chart provided by the manufacturer was used to interpret the result.

### 2.6. HIV Screening and Monitoring Assays

HIV serostatus of participants was determined using HIV-1 rapid assays based on the national HIV testing algorithm. CD4+ T-cell number and plasma HIV-1 RNA viral load were quantified for the participants with HIV for this research purpose. Fresh blood collected with EDTA tubes were used for CD4+ T-cell count determination on FACSCalibur machine (BD bioscience). Plasma separated from the remaining EDTA blood was then used to measure the plasma HIV-1 RNA viral load on Cobas Amplipre/Taqman automated real-time PCR (Abbott Laboratories). The assay detection limit of the viral load assay is 48–10^7^ copies/ml and results below 20 copies/ml report as lower detection limit (LDL).

### 2.7. Data Analysis

Statistical Package for Social Science version 20.0 (SPSS, IBM, Armonk, USA) and GraphPad Prism 6.0 (GraphPad Software, La Jolla California, USA) were used for statistical data analyses and generating graphs, respectively. Group comparison of TLRs expression was made using nonparametric Kruskal–Wallis test followed by Dunn's multiple comparison test for intergroup comparisons. On the graphs, median values were presented as lines within dotplots. Correlations of variables were assessed using the nonparametric Spearman correlation test. Correlations were presented as a correlation coefficient (*r*) along with *p*-values. Results were considered statistically significant with *p*-values (*p*) less than 0.05.

## 3. Results

### 3.1. Participants Demographic and Clinical Characteristics

Most of the participants were of similar age with a median of 30 years with inter quartile range of 26–36 years, although the TB/HIV group was slightly older with median age of 41 years. The male:female ratio was 1.2. The majority of participants with TB were diagnosed with pulmonary TB. Upon enrollment, 84% of the HIV group presented with WHO clinical stage I or II, while all of the participants in the TB/HIV group presented with clinical stage III or IV. Majority of participants with HIV had detectable viral load, with a median of 52,771 (IQR: 1−18 × 10^4^) and 360,627 (IQR: LDL−2×10^6^) copies/ml for HIV and TB/HIV, respectively. From the 14 plasma markers measured, only eight of them had an acceptable number of samples (>50%) with a quantifiable result. TB group had higher plasma levels of CCL3, CCL4, IP10, TNF*α*, IL6, IFN*γ*, and IL10 compared to HC. Likewise, the HIV group had higher levels of TNF*α*, IP10, IFN*γ*, and IL10 compared to HC. The TB/HIV group had the higher level of most plasma markers (Table 1).

### 3.2. TLR2 and TLR4 but not TLR9 Increased in TB and HIV

**Figure 1:**
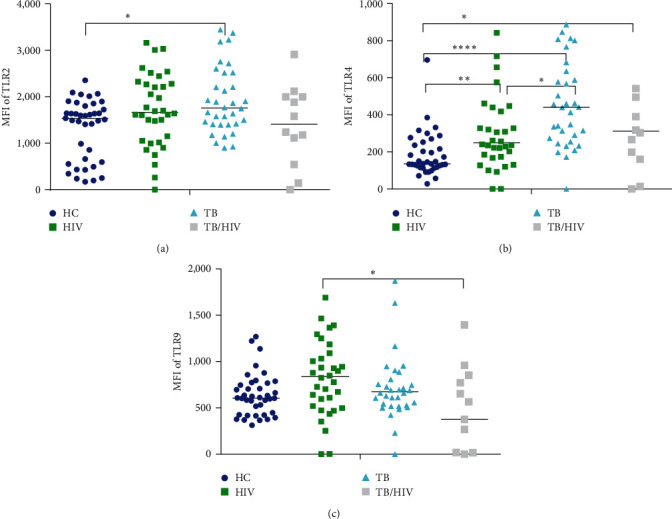
TLR2, TLR4, and TLR9 expression on CD14+ monocytes. Graphs represent expression of (a) TLR2, (b) TLR4, and (c) TLR9 in HC, HIV, TB, and TB/HIV. *Y*-axis represents the median fluorescence intensity (MFI) of the markers and *X*-axis represents the study participant group. The midlines in the dotplots represent the median values. Comparison of markers between the participant group was made with the nonparametric Kruskal–Wallis test followed by Dunn's multiple comparison was used. The asterisks represent *p*-values of less than  ^*∗*^0.05,  ^*∗∗*^0.01, and  ^*∗∗∗∗*^0.0001.

**Figure 2:**
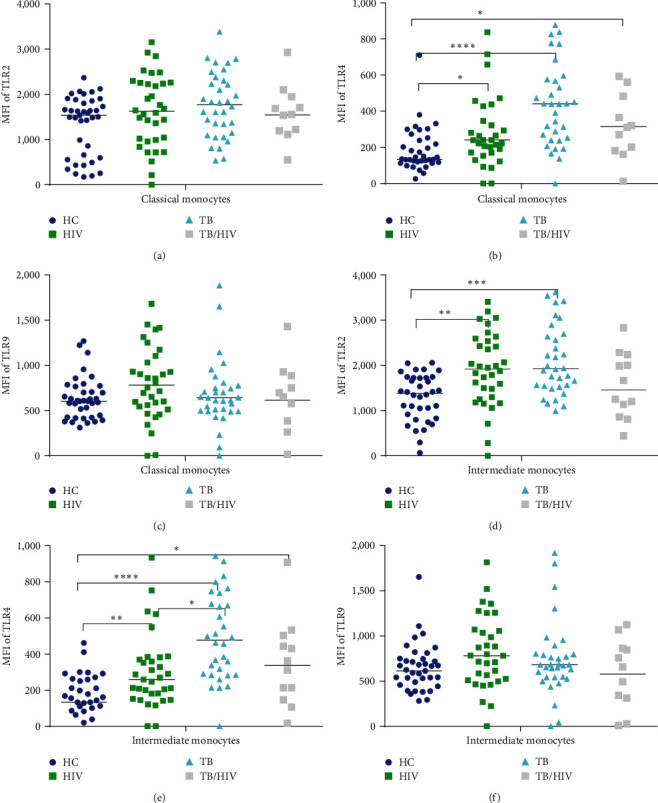
TLR2, TLR4, and TLR9 expression on classical and intermediate monocytes. *Y*-axis represents the MFI of the markers and *X*-axis represents the monocyte subsets: (a–c) classical monocytes, and (d–f) intermediate monocytes. The midlines in the dotplots represent the median values. Comparison of markers between the participant group was made with the nonparametric Kruskal–Wallis test followed by Dunn's multiple comparison was used. The asterisks represent *p*-values of less than  ^*∗*^0.05,  ^*∗∗*^0.01,  ^*∗∗∗*^0.001, and  ^*∗∗∗∗*^0.0001.

The MFI of TLR2 on CD14+ monocytes was significantly higher in TB compared to HC, *p* < 0.05 (Figure 1(a)). The expression intensity of TLR2 was also slightly increased in HIV. Interestingly, expression of TLR2 in TB/HIV was slightly lower compared to its expression on HC. However, the change in TLR2 expression in HIV and TB/HIV did not reach statistical significance. Our findings showed higher density of TLR2 expressed on IM in TB (*p* < 0.0001) and HIV (*p* < 0.01) compared to TLR2 expressed on IM in HC. We also observed similar greater expression of TLR2 on CM in TB than HC (Figures 2(a) and 2(d)). Intradisease comparison of TLR2 expression between CM and IM showed a greater expression of TLR2 on IM than CM, but this did not reach statistical significance.

Figure 1(b) illustrates TLR4 expression on CD14+ monocytes, which was significantly higher in TB (*p* < 0.0001), HIV (*p* < 0.01), and TB/HIV (*p* < 0.05) than HC. Furthermore, the intensity of TLR4 on CD14+ monocytes in TB was also higher than its expression in HIV (*p* < 0.05). In an attempt to assess the TLR4 expression difference between CM and IM subsets, our findings indicated that the expression of TLR4 was significantly elevated on both subsets in all patient groups compared to expression on respective monocyte subsets in HC (Figures 2(b) and 2(e)). Thus, the intensity of TLR4 on CM in TB, HIV, and TB/HIV was higher compared to HC, *p* < 0.0001, 0.05, and 0.05, respectively. Likewise, TLR4 expression on IM in TB, HIV, and TB/HIV was higher compared to HC, *p* < 0.0001, 0.01, and 0.05, respectively. Yet, expression of TLR4 between CM and IM within a study group was not statistically significant.

There was a slight increase in the expression of TLR9 in TB and HIV while a slight reduction in TB/HIV compared to HC but none of it reached statistical significance (Figure 1(c)). Interestingly, the difference between HIV and TB/HIV was statistically significant (*p* < 0.05). Neither disease specific nor monocyte subset specific comparisons of TLR9 showed a statistically significant difference in expression (Figures 2(c) and 2(f)).

We performed QuantiFERON TB Gold+ test to determine TB infection status of HC. The overall QuantiFERON positivity (latent Tuberculosis infection-LTBI) was 40%. We compared TLRs expression between QuantiFERON positive and QuantiFERON negative HC and did not observe statistically significant difference though there was a slight increase in TLR expression in the LTBI group (data not shown).

### 3.3. Correlation of TLRs with Clinical Indices

Different clinical indices were used as proxy indicators to measure disease severity. Thus, we correlated TLR expression with these clinical indicators (details of the correlation are provided in *Supplementary 2*; Table 2). Our results demonstrated that TLR2 expressed on CD14+ monocytes had a positive correlation with body mass index (BMI) in HC, *r* = 0.453; *p* < 0.01, but not in the diseased groups. As CD4 count results were missing for some of the participants in both the HIV and TB/HIV groups, we combined the CD4 data of the two cohorts and performed correlation analysis. The result revealed that TLR2 positively correlated with CD4 count, *r* = 0.354; *p* < 0.05. On the other hand, HIV-1 viral load inversely correlated with TLR2 (*r* = −0.357; *p* < 0.05) while positively correlated with TLR4 (*r* = 0.578; *p* < 0.05) and TLR9 (*r* = 0.655; *p* < 0.05). Interestingly, none of the TLRs in any of the study groups had a significant correlation with mycobacterial indices, AFB grade and LAM.

### 3.4. Correlation of TLRs with Plasma Cytokines

**Figure 3:**
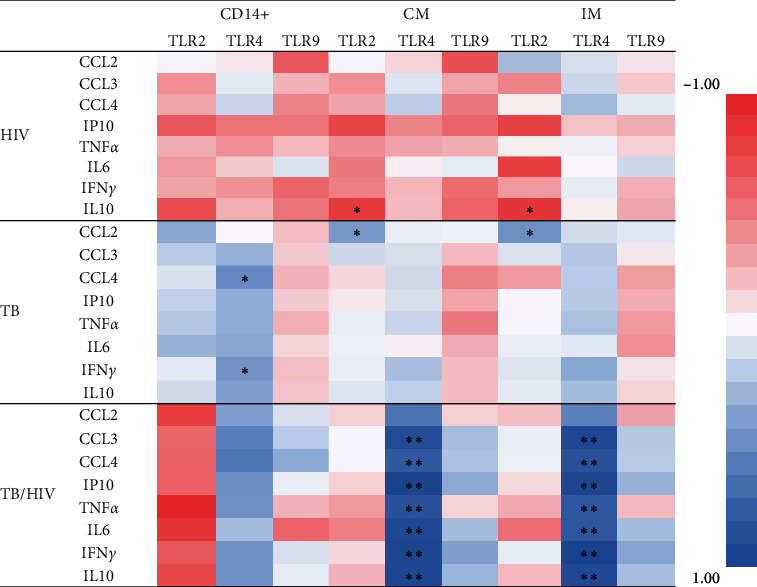
Correlations of surface markers with plasma biomarkers. *Note*. Heat map represents coefficient of variation (*r*), asterisks represent *p*-values of <^*∗*^0.05 and <^*∗∗*^0.01.

Figure 3 provides the results of the correlation analysis of TLRs with plasma biomarkers. In HC, TLR2 and TLR4 expressed on CD14+ monocytes had a positive correlation with plasma IL6 level *r* = 0.648; *p* < 0.05 and *r* = 0.636; *p* < 0.05, respectively. Conversely, TLR4 had a negative correlation pattern with IL10 (*r* = −0.572; *p* < 0.07). Surprisingly, the expression of TLRs on CD14+ monocytes in all patient groups did not significantly correlate with any of the cytokines except for TLR4 with CCL4 (*r* = 0.482; *p* < 0.05) and IFN*γ* (*r* = 0.417; *p* < 0.05) in TB. When the data were disaggregated into CM and IM subsets, the correlation became more evident. In the HIV group, TLR2 expressed on IM were correlated with IL6 (*r* = −0.428; *p* < 0.05), IP10 (*r* = −0.415; *p* < 0.05), and IL10 (*r* = −0.541; *p* < 0.05). Similarly, TLR2 expressed on CM were correlated with IP10 (*r* = −0.406; *p* < 0.05) and IL10 (*r* = −0.433; *p* < 0.05). In TB/HIV, TLR4 expressed on IM had positive correlation with CCL2 (*r* = 0.606; *p* < 0.05), CCL3 (*r* = 0.803; *p* < 0.01), TNF*α* (*r* = 0.722; *p* < 0.01), IL6 (*r* = 0.658; *p* < 0.05), IFN-*γ* (*r* = 0.849; *p* < 0.0001), IP10 (*r* = 0.739; *p* < 0.01), and IL10 (*r* = 0.760; *p* < 0.01). Similarly, all the aforementioned correlations between TLR4 and the chemokines/cytokines in TB/HIV were also valid in CM subset. Details of the correlation are provided in *Supplementary 2*.

## 4. Discussion

Previous studies have evaluated levels of TLR mRNA in diseased participants [7, 9, 26–28]. We report here the protein expression of TLRs on cells in patients with HIV and TB/HIV coinfection. We evaluated the density of TLR on monocyte subsets in patients with TB, HIV, and TB/HIV coinfection, and also assessed their correlation with clinical status and plasma cytokines. While no associations with clinical diagnosis, status, or cytokines/chemokines were observed with TLR9, there were several differences seen with TLR4 and TLR2.

TLR4 was significantly enhanced relative to HC in HIV, TB, and TB/HIV coinfected patients, with the greatest levels seen in participant with TB, which were more than fourfold higher relative to HC. Enhancements were seen in both classical and intermediate monocyte subsets. There were no correlations of TLR4 with indicators of clinical severity among participants with HIV or TB. Interestingly, however, we did observe significant correlations between the levels of TLR4 and several cytokines and chemokines among participants with TB/HIV coinfection. The mechanisms underlying both the apparent disease-specific changes in the expression of TLR4 as well as their associations with cytokine production in some patients are not clear.

Several factors may influence TLR levels and could be impacting results here. More generally, we think of microbial products or PAMPs as acting directly on TLR and inducing the production of inflammatory mediators such as cytokines [5–7]; evidence of dysregulation or function of these pathways have been described in HIV and/or TB disease [7, 9, 29]. However, there is also evidence that TLR agonists in addition to inducing signal transduction, also directly modulate TLR levels. For example, lipopolysaccharide (LPS), while upregulating TLR4 mRNA, actually contributes to its surface endocytosis and downregulation [30]. Similarly, while cytokines may be induced by TLR signaling, they may also feedback and directly modulate TLR levels. IFNs and granulocyte monocyte colony-stimulating factor (GM-CSF), for example, is known to upregulate TLR2 and TLR4 levels [31, 32], and hormones are known as well to impact TLR expression [33]. These latter studies have not been performed in patients with TB or HIV, but in light of our findings here, future *in vitro* work along these lines may be informative to elucidate underlying mechanisms.

TLR2, like TLR4, was enhanced in participants with TB or HIV, but not so among participants with TB/HIV coinfection, pointing to disease-specific factors modulating expression. Moreover, the disease association was primarily seen in the intermediate monocyte subset. One possible reason for the reduced TLR2 and TLR9 in our participants with TB/HIV coinfection could be that HIV diminishes TLRs expression and functionality. Few studies have reported that HIV may hinder immune cell responses to TLR ligands [34, 35]. PBMCs from HIV showed reduced responsiveness to TLR agonists compared to PBMCs from HC [36]. In part, such reduction is attributed to the direct interference of HIV antigen on TLRs. One such protein is the HIV accessory protein, Vpu. This accessory protein antagonizes TLR7 and TLR9 signaling in plasmacytoid DCs. Yet, the exact mechanism of how HIV reduces responsiveness to TLR agonists is not known [7]. An alternate reason for the reduction of TLR expression in patients with TB/HIV coinfection could be immune deterioration/immune exhaustion [37, 38] as most of our TB/HIV patients were in an advanced disease state. Although we consider it is unlikely that the observed differences in TLRs expression between TB/HIV coinfected patients in comparison with HIV or TB monoinfected patients was related to the use of ART, we cannot exclude this possibility as our coinfected subjects had mixed ART status.

Though the correlation was weak, a positive correlation of TLR2 with CD4 count in patients with HIV while having an inverse correlation with HIV-1 viral load, as well as the positive correlation of TLR2 with BMI observed in HC strengthens the above speculation. The observed correlations of TLRs with measurable clinical markers were also evident when we categorized patients into different groups based on their clinical stage and immunocompetency. When participants were categorized into early (clinical stages I and II) and late (clinical stages III and IV) HIV infection, participants at early stage of HIV had higher TLR2 and TLR9 than participants at late stage of HIV infection (data not shown). In the clinically categorized group, the late HIV group had higher TLR4 than the early HIV group. In addition, although it was not statistically significant, people with HIV who had CD4 count more than 500 cell/mm^3^ had higher TLR2 and TLR9 levels than those with CD4 count less than 500 cell/mm^3^. Taking together, this may imply TLR2 expression is associated with immunocompetent status while TLR4 expression is with disease progression and immunologic dysfunction/exhaustion.

Consistent with the above possibility, we also observed that TLR2 levels had inverse association pattern with serum cytokines in participants with HIV, known to be elevated and related to disease progression, and this association was seen in both classical and intermediate monocytes. Thus, there appear to be some disease-specific factors, perhaps cytokines, involved in TLR2 modulation particularly in advanced disease, but also an additional mechanism responsible for the upregulation of TLR2, and this is prominently seen in the intermediate monocyte subset.

The observation that TLR2 and TLR4 showed different patterns of association with cytokines, as well as different levels in intermediate and classical monocyte subsets, reinforces the likelihood that multiple underlying factors are contributing to the TLR patterns. For example, while both TLR2 and TLR4 initiate signaling through the MyD88-dependent signaling, TLR4 can also signal independently of MyD88 [1]. *In vitro* and *in vivo* studies demonstrated that intermediate monocytes produce high level of proinflammatory cytokines such as IL1*β*, TNF*α*, and IL6 [39–41] thus it is more likely that TLRs expressed on intermediate monocytes to correlate with inflammatory cytokines. *In vitro* studies on PBMCs and alveolar macrophages demonstrated differential induction of cytokines in response to TLR agonists [28, 42]. Stimulation of alveolar macrophages with several responding TLR agonists resulted in strong TLR4-dependent immune response with massive cytokine and chemokine genes expression (CCL2, CCL3, CCL4, CXCL10, TNF*α*, IL6, and IL10) while moderate level TLR2 dependent immune response with fewer gene expressions but weaker TLR9 dependent responses [42]. In another study, PBMCs stimulated with TLR2, TLR4, and TLR9 agonists along with HIV-1 virus resulted in the induction of TNF*α* and IL6 mRNA in TLR4-dependent manner but reduced IL6 mRNA in TLR2 and TLR9 based activation [28].

The higher levels of both TLR2 and TLR4 on intermediate monocyte are similar to our previous findings of higher chemokine receptors [43] and this may relate to the proposed antigen-presenting function of this subset [40]. Intermediate monocytes express high levels of MHC II genes as well as proteins (e.g., HLADR), and costimulatory molecules such as CD86 and CD40 [39, 40, 44]. Few studies previously reported higher expression of TLRs on CD16+ monocytes than CD16− monocyte subsets in infectious and noninfectious inflammatory diseases [45, 46]. Quixabeira et al. [46] demonstrated overall increase in TLR2 and TLR4 expression on the three monocytes subsets in *Cutaneous leishmaniosis* patients than healthy controls with higher TLR2 but not TLR4 expressed on IM than CM. The group also demonstrated nonclassical monocytes are the least expressers of both TLR2 and TLR4. In rheumatoid arthritis patients, CD16+ monocytes expressed higher TLR2 than CD16− monocytes while no TLR4 expression level difference between the two monocyte subsets [45]. Taking together, the enhanced expressions of TLRs, chemokine receptors, adhesion molecules, antigen presenting, and costimulatory molecules, as well as high inflammatory cytokine production by intermediate monocytes represents this subset is the highly activated phenotype of the subsets. The other two subsets of monocytes, classical and nonclassical monocytes mainly have phagocytic and patrolling/tissue repair roles, respectively [39, 40].

Nonclassical monocytes were not evaluated in this study due to inability to resolve them clearly from natural killer cells with the markers we used; future experiments will address this. In addition, we cannot exclude the possibility that some of the observed effects were influenced by other concurrent unknown microbial pathogens.

In summary, we have demonstrated that TLR expression on monocytes in TB, HIV, and TB/HIV are correlated with plasma biomarkers. Thus, we have demonstrated for the first time (i) protein level expression of TLRs in HIV and TB/HIV, (ii) differential expression of TLRs in CM and IM subsets in TB, HIV, and TB/HIV coinfection, (iii) that expression of TLRs varies as the diseases progress, and (iv) TLR expression differentially correlate with mycobacterial and host indices.

## Figures and Tables

**Table 1:** Study participants demographic and clinical characteristics.

Variables	HC (*n* = 39)	HIV (*n* = 35)	TB (*n* = 34)	TB/HIV (*n* = 12)
Age in years, median	28	33	30	41
Male sex at birth (%)	58.3	42.4	69.2	50
Body mass index	21.9	20.6	18.3	19.6
BCG scar present (%)	42	20	40	42
PTB (%)	N/A	N/A	89	70
HIV clinical stages I and II (%)	N/A	83.3	N/A	0
HIV viral load, median (IQR)	N/A	5.3 × 10^4^ (1–18 × 10^4^)	N/A	3.6 × 10^5^ (LDL^#^−2 × 10^6^)
Absolute CD4, median (IQR)	N/A	270 (94–599) ^*∗*^	N/A	210 (85–289) ^*∗∗*^
CCL2^ǂ^	129	148	114	136
CCL3	UD	UD	135	UD
CCL4	UD	UD	138	79
CXCL10 (IP10)	39	107	234	125
TNF*α*	1.2	2.4	2.7	2.4
IFN*γ*	UD	2.75	12.5	3.69
IL6	0.69	0.53	7.66	2.45
IL10	1.6	6.14	19.1	7.37

^ǂ^Plasma biomarkers in ug/ml and UD, undetectable, ^#^48 copies/ml,  ^*∗*^sample size of 24, and  ^*∗∗*^sample size of 7.

**Table 2:** Correlations of toll-like receptors with clinical indices in CD14+ monocytes, classical, and intermediate monocyte subsets.

Markers		BMI					CD4			HIV-1 viral load		
		ALL	HC	TB	HIV	TB/HIV	ALL	HIV	TB/HIV	ALL	HIV	TB/HIV
CD14+	TLR2	0.036	0.453 ^*∗∗*^	−0.112	0.172	−0.257	0.353^*∗*^	0.297	0.481	−0.169	−0.218	−0.077
	TLR4	−0.252^*∗*^	−0.002	−0.267	0.115	−0.429	0.134	0.091	0.493	−0.107	0.261	0.042
	TLR9	−0.035	−0.299	0.281	0.263	−0.029	0.178	0.075	0.22	−0.021	−0.113	0.282

CM	TLR2	0.099	0.363^*∗*^	−0.051	0.201	−0.257	0.404 ^*∗∗*^	0.331	0.593^*∗*^	−0.19	−0.255	0.007
	TLR4	−0.242^*∗*^	−0.029	−0.272	0.112	−0.486	0.032	0.03	0.149	0.063	−0.221	0.521
	TLR9	−0.052	−0.217	0.274	0.31	−0.829^*∗*^	0.085	0.092	−0.172	0.16	−0.111	0.704 ^*∗∗*^

IM	TLR2	−0.072	0.169	−0.054	0.045	−0.314	0.234	0.103	0.444	−0.238	−0.357^*∗*^	0.12
	TLR4	−0.331 ^*∗∗*^	−0.064	−0.307	−0.001	−0.371	0.034	−0.047	0.294	0.095	−0.129	0.578^*∗*^
	TLR9	−0.143	−0.345	0.147	0.205	−0.886^*∗*^	−0.021	−0.091	−0.131	0.157	−.076	.655^*∗*^

ALL means total study participants (*n* = 120), asterisks represent *p*-value of less than  ^*∗*^0.05,  ^*∗∗*^0.01.

## Data Availability

Raw data will be available upon request to the corresponding author.

## Supplementary Materials

Supplementary 1 Gating strategy for CD14+ monocytes, their subsets and markers antibody-isotype difference. First, doublets were gated out based on their position in the forward light scatter (FSC)-Area versus FSC-Height plot. Then FL8 channel was used as a damp channel to detect and eliminate non-specific fluorescing cells as no flourochrome staining included for this channel. CD14+ monocyte enriched populations were gated based on CD14 positivity. CD16 positivity was used to further sub-classify classical (CM) and intermediate (IM) monocytes subsets. Then, the median fluorescence intensity (MFI) of TLR2, TLR4 and TLR9 antibodies and corresponding isotype controls were measured on total CD14+ monocytes, CM and IM subsets. Finally, net MFI (nMFI) of TLRs calculated by subtracting the MFI of isotype controls from MFI of corresponding TLR antibodies.

Supplementary 2 Protein expression of TLR2, TLR4 and TLR9 on CD14+ monocytes, classical and intermediate monocytes in TB, HIV and TB/HIV: *Supplementary* 2 provided multiple correlations of plasma chemokines and cytokines with TLR2, TLR4 and TLR9 expressed on CD14+ monocytes, classical and intermediate monocyte subsets in each study cohort HC, HIV, TB and TB/HIV.
