# Supplementary material for: Protein Expression of TLR2, TLR4, and TLR9 on Monocytes in TB, HIV, and TB/HIV
Source: J Immunol Res. 2024 Apr 17;2024:9399524. doi: 10.1155/2024/9399524 (PMC11042910; doi:10.1155/2024/9399524)
Supplement: Supplementary 1 — Gating strategy for CD14+ monocytes, their subsets and markers antibody-isotype difference. First, doublets were gated out based on their position in the forward light scatter (FSC)-Area versus FSC-Height plot. Then FL8 channel was used as a damp channel to detect and eliminate non-specific fluorescing cells as no flourochrome staining included for this channel. CD14+ monocyte enriched populations were gated based on CD14 positivity. CD16 positivity was used to further sub-classify classical (CM) and intermediate (IM) monocytes subsets. Then, the median fluorescence intensity (MFI) of TLR2, TLR4 and TLR9 antibodies and corresponding isotype controls were measured on total CD14+ monocytes, CM and IM subsets. Finally, net MFI (nMFI) of TLRs calculated by subtracting the MFI of isotype controls from MFI of corresponding TLR antibodies. [file 9399524.f1.docx]

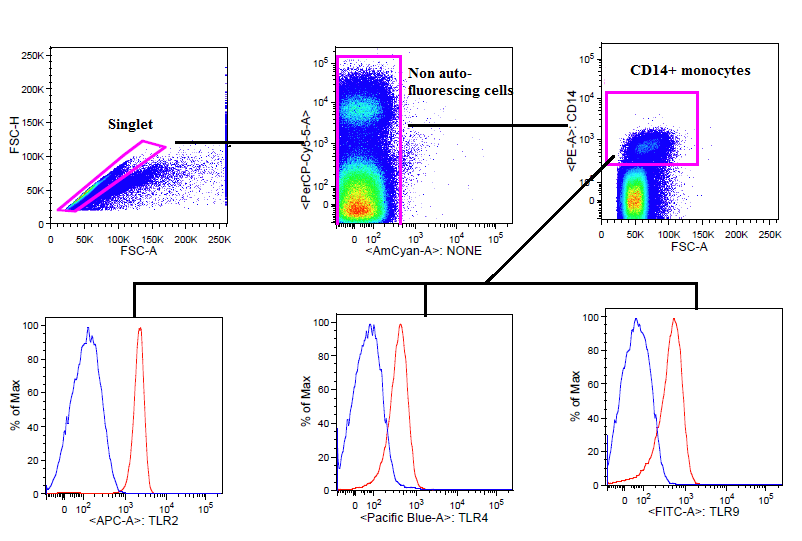
***Supplementary figure 1: Gating strategy for CD14+ monocytes, their subsets and markers antibody-isotype difference.*** *First, doublets were gated out based on their position in the forward light scatter (FSC)-Area versus FSC-Height plot. Then FL8 channel was used as a damp channel to detect and eliminate non-specific fluorescing cells as no flourochrome staining included for this channel. CD14+ monocyte enriched populations were gated based on CD14 positivity. CD16 positivity was used to further sub-classify classical (CM) and intermediate (IM) monocytes subsets. Then, the median fluorescence intensity (MFI) of TLR2, TLR4 and TLR9 antibodies and corresponding isotype controls were measured on total CD14+ monocytes, CM and IM subsets. Finally, net MFI (nMFI) of TLRs calculated by subtracting the MFI of isotype controls from MFI of corresponding TLR antibodies.*
