# Supplementary material for: Protein Expression of TLR2, TLR4, and TLR9 on Monocytes in TB, HIV, and TB/HIV
Source: J Immunol Res. 2024 Apr 17;2024:9399524. doi: 10.1155/2024/9399524 (PMC11042910; doi:10.1155/2024/9399524)
Supplement: Supplementary 2 — Protein expression of TLR2, TLR4 and TLR9 on CD14+ monocytes, classical and intermediate monocytes in TB, HIV and TB/HIV: Supplementary 2 provided multiple correlations of plasma chemokines and cytokines with TLR2, TLR4 and TLR9 expressed on CD14+ monocytes, classical and intermediate monocyte subsets in each study cohort HC, HIV, TB and TB/HIV. [file 9399524.f2.doc]

**Supplementary tables: Protein expression of TLR2, TLR4 and TLR9 on monocytes in TB, HIV and TB/HIV**

**Supplementary Table 1.** Correlation of plasma biomarkers with TLR expression in HC, HIV, TB and TB/HIV

**Table 1.1** Correlation of plasma biomarkers with TLR expression in HC

|  | | CD14+ | | | CM | | | IM | | |
| --- | --- | --- | --- | --- | --- | --- | --- | --- | --- | --- |
|  | | TLR2 | TLR4 | TLR9 | TLR2 | TLR4 | TLR9 | TLR2 | TLR4 | TLR9 |
| CCL2 | r | .355 | -.209 | -.027 | .355 | -.055 | -.014 | -.127 | .200 | .036 |
|  | p | .285 | .537 | .937 | .285 | .873 | .968 | .726 | .580 | .915 |
| CCL3 | r |  |  |  |  |  |  |  |  |  |
|  | p |  |  |  |  |  |  |  |  |  |
| CCL4 | r | .290 | -.174 | -.290 | .290 | -.058 | -.291 | -.548 | .137 | -.174 |
|  | p | .416 | .631 | .416 | .416 | .873 | .415 | .127 | .725 | .631 |
| IFNg | r |  |  |  |  |  |  |  |  |  |
|  | p |  |  |  |  |  |  |  |  |  |
| IL10 | r | .009 | -.572 | -.395 | .009 | -.284 | -.413 | -.252 | -.448 | -.014 |
|  | p | .978 | .066 | .229 | .978 | .398 | .207 | .483 | .194 | .968 |
| IL6 | r | **.648*** | **.636*** | -.248 | **.648*** | .382 | -.213 | .600 | .567 | -.370 |
|  | p | **.043** | **.048** | .489 | **.043** | .276 | .555 | .088 | .112 | .293 |
| IP10 | r | .127 | -.509 | -.555 | .127 | -.264 | -.565 | -.018 | -.442 | -.100 |
|  | p | .709 | .110 | .077 | .709 | .433 | .070 | .960 | .200 | .770 |
| TNFa | r | .303 | -.395 | -.524 | .303 | -.106 | -.548 | .110 | -.366 | -.179 |
|  | p | .364 | .229 | .098 | .364 | .757 | .081 | .763 | .298 | .598 |
| r = coefficient of variation, p= p-value. *. Correlation is significant at the 0.05 level (2-tailed). | | | | | | | | | | |

**Supplementary Table 1.** Correlation of plasma biomarkers with TLR expression in HC, HIV, TB and TB/HIV

**Table 1.2** Correlation of plasma biomarkers with TLR expression in HIV

|  | | CD14+ | | | CM | | | IM | | |
| --- | --- | --- | --- | --- | --- | --- | --- | --- | --- | --- |
|  | | TLR2 | TLR4 | TLR9 | TLR2 | TLR4 | TLR9 | TLR2 | TLR4 | TLR9 |
| CCL2 | r | -.046 | -.074 | -.350 | -.028 | -.099 | -.368 | .221 | .064 | -.080 |
|  | p | .821 | .715 | .074 | .893 | .630 | .064 | .278 | .755 | .699 |
| CCL3 | r | -.222 | .034 | -.156 | -.219 | .038 | -.177 | -.246 | .090 | -.122 |
|  | p | .265 | .866 | .437 | .282 | .852 | .386 | .226 | .664 | .553 |
| CCL4 | r | -.181 | .101 | -.246 | -.184 | .136 | -.268 | -.064 | .237 | .030 |
|  | p | .387 | .630 | .237 | .390 | .525 | .205 | .767 | .265 | .890 |
| IFNg | r | -.186 | -.219 | -.314 | -.254 | -.150 | -.296 | -.204 | .013 | -.160 |
|  | p | .362 | .283 | .118 | .220 | .473 | .151 | .329 | .949 | .444 |
| IL10 | r | -.377 | -.163 | -.276 | **-.433*** | -.143 | -.315 | **-.451*** | -.063 | -.176 |
|  | p | .053 | .416 | .163 | **.027** | .486 | .117 | **.021** | .760 | .391 |
| IL6 | r | -.207 | -.116 | .048 | -.267 | -.058 | .018 | **-.428*** | -.041 | .087 |
|  | p | .301 | .565 | .813 | .188 | .779 | .931 | **.029** | .841 | .674 |
| IP10 | r | -.351 | -.279 | -.279 | **-.406*** | -.244 | -.312 | **-.415*** | -.131 | -.164 |
|  | p | .073 | .159 | .159 | **.040** | .229 | .121 | **.035** | .525 | .424 |
| TNFa | r | -.164 | -.224 | -.145 | -.227 | -.180 | -.164 | -.063 | -.023 | -.109 |
|  | p | .413 | .262 | .470 | .265 | .379 | .423 | .758 | .910 | .598 |
| r = coefficient of variation, p= p-value. *. Correlation is significant at the 0.05 level (2-tailed). | | | | | | | | | | |

**Supplementary Table 1.** Correlation of plasma biomarkers with TLR expression in HC, HIV, TB and TB/HIV

**Table 1.3** Correlation of plasma biomarkers with TLR expression in TB

|  | | CD14+ | | | CM | | | IM | | |
| --- | --- | --- | --- | --- | --- | --- | --- | --- | --- | --- |
|  | | TLR2 | TLR4 | TLR9 | TLR2 | TLR4 | TLR9 | TLR2 | TLR4 | TLR9 |
| CCL2 | r | .328 | -.044 | -.137 | **.400*** | .003 | -.010 | **.442*** | .072 | .035 |
|  | p | .088 | .823 | .487 | **.035** | .989 | .960 | **.019** | .717 | .860 |
| CCL3 | r | .159 | .287 | -.118 | .087 | .065 | -.143 | .048 | .160 | -.071 |
|  | p | .459 | .174 | .584 | .686 | .763 | .505 | .822 | .454 | .743 |
| CCL4 | r | .050 | **.482*** | -.157 | -.095 | .083 | -.250 | -.201 | .145 | -.196 |
|  | p | .811 | **.015** | .454 | .653 | .694 | .227 | .334 | .489 | .347 |
| IFNg | r | .024 | **.417*** | -.135 | -.001 | .205 | -.139 | .040 | .334 | -.075 |
|  | p | .907 | **.034** | .512 | .996 | .315 | .499 | .845 | .096 | .716 |
| IL10 | r | .077 | .357 | -.131 | .037 | .130 | -.140 | .027 | .219 | -.105 |
|  | p | .704 | .068 | .514 | .856 | .517 | .485 | .892 | .272 | .602 |
| IL6 | r | .267 | .329 | -.097 | -.002 | -.055 | -.165 | .003 | .036 | -.219 |
|  | p | .170 | .087 | .622 | .990 | .780 | .401 | .988 | .857 | .263 |
| IP10 | r | .120 | .305 | -.116 | -.072 | .059 | -.183 | -.048 | .152 | -.165 |
|  | p | .542 | .115 | .557 | .715 | .765 | .352 | .810 | .441 | .402 |
| TNFa | r | .166 | .309 | -.162 | .002 | .104 | -.266 | -.029 | .198 | -.200 |
|  | p | .399 | .110 | .409 | .990 | .597 | .172 | .882 | .312 | .307 |
| r = coefficient of variation, p= p-value. *. Correlation is significant at the 0.05 level (2-tailed). | | | | | | | | | | |

**Supplementary Table 1.** Correlation of plasma biomarkers with TLR expression in HC, HIV, TB and TB/HIV

**Table 1.4** Correlation of plasma biomarkers with TLR expression in TB/HIV

|  | | CD14+ | | | CM | | | IM | | |
| --- | --- | --- | --- | --- | --- | --- | --- | --- | --- | --- |
|  | | TLR2 | TLR4 | TLR9 | TLR2 | TLR4 | TLR9 | TLR2 | TLR4 | TLR9 |
| CCL2 | r | -.427 | .355 | .055 | -.109 | .600 | -.109 | -.136 | .533 | -.191 |
|  | p | .190 | .285 | .873 | .750 | .051 | .750 | .689 | .091 | .574 |
| CCL3 | r | -.311 | .548 | .146 | -.027 | **.886**** | .215 | .005 | **.913**** | .169 |
|  | p | .353 | .081 | .668 | .936 | **.000** | .526 | .989 | **.000** | .619 |
| CCL4 | r | -.323 | .592 | .319 | -.032 | **.779**** | .200 | -.005 | **.833**** | .155 |
|  | p | .332 | .055 | .339 | .926 | **.005** | .555 | .989 | **.001** | .649 |
| IFNg | r | -.351 | .428 | .055 | -.096 | **.934**** | .364 | .023 | **.966**** | .337 |
|  | p | .290 | .189 | .873 | .780 | **.000** | .270 | .947 | **.000** | .311 |
| IL10 | r | -.391 | .436 | .018 | -.155 | **.927**** | .255 | -.145 | **.907**** | .209 |
|  | p | .235 | .180 | .958 | .650 | **.000** | .450 | .670 | **.000** | .537 |
| IL6 | r | -.445 | .218 | -.318 | -.255 | **.918**** | .218 | -.291 | **.802**** | .218 |
|  | p | .170 | .519 | .340 | .450 | **.000** | .519 | .385 | **.003** | .519 |
| IP10 | r | -.327 | .455 | 0.000 | -.109 | **.955**** | .318 | -.091 | **.934**** | .273 |
|  | p | .326 | .160 | 1.000 | .750 | **.000** | .340 | .790 | **.000** | .417 |
| TNFa | r | -.518 | .445 | -.155 | -.209 | **.836**** | -.100 | -.173 | **.797**** | -.145 |
|  | p | .102 | .170 | .650 | .537 | **.001** | .770 | .612 | **.003** | .670 |
| r = coefficient of variation, p= p-value. **. Correlation is significant at the 0.01 level (2-tailed). | | | | | | | | | | |
